# How do African elephants utilize the landscape during wet season? A habitat connectivity analysis for Sioma Ngwezi landscape in Zambia

**DOI:** 10.1002/ece3.8177

**Published:** 2021-10-07

**Authors:** Doubt Chibeya, Heather Wood, Sara Cousins, Kerryn Carter, Moses Amos Nyirenda, Henry Maseka

**Affiliations:** ^1^ Biogeography and Geomatics Department of Physical Geography Stockholm University Stockholm Sweden; ^2^ Elephant Connection Research Project Lusaka Zambia; ^3^ World Wide Fund for Nature‐Zambia Lusaka Zambia; ^4^ Department of National Parks and Wildlife Lusaka Zambia

**Keywords:** habitat suitability, human–elephant conflicts, KAZA, *Loxodonta africana*, maximum entropy, resistant raster, telemetry data

## Abstract

1. The influence of environmental factors on the distribution and persistence of African elephants (*Loxodonta africana*) is pertinent to policy makers and managers to formulate balanced plans for different land‐use types.
2. The study focuses on movement of elephants and how they utilize foraging areas in Sioma Ngwezi landscape in Zambia by answering the following questions: (1) Which environmental variables and land‐cover class predict the movement of elephants during the wet season in Sioma Ngwezi landscape? (2) What is the wet season suitable habitat for elephants in Sioma Ngwezi landscape? (3) What are the major wet season movement corridors for elephants in Sioma Ngwezi landscape?
3. We used GPS telemetry data from the collared elephants to assess habitat connectivity. Maximum entropy (MaxEnt) and linkage mapper were the tools used to predict habitat suitability, movement corridors, and barriers in the landscape during the wet season.
4. The study identified elevation, land cover, and NDVI as the most important environmental predictors that modify the dispersal of elephants in the landscape during the wet season. Additionally, a total of 36 potential wet season corridors were identified connecting 15 core areas mainly used for foraging and protection from poachers in the landscape. Of these, 24 corridors were highly utilized and are suggested as priority corridors for elephant movement in the landscape.
5. The identified wet season habitats and functional corridors may help to combat elephant poaching by patrolling areas with high relative probability of elephant presence. The findings may also help abate human–elephant conflict such as crop‐raiding by managing identified corridors that run into agriculture zones in the game management area.

## Graphical abstract

The study focuses on movement of elephants and how they utilize foraging areas in wet season in Sioma Ngwezi landscape in Zambia.

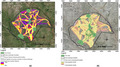

## 1 INTRODUCTION

The integrity and functionality of an ecosystem coupled with its biodiversity and ecosystem services maintenance is made possible by the flow of species, materials, energy, and information across the landscape (Ayram et al., 2016). This knowledge has awakened serious interest in conservation biology especially landscape connectivity studies. Connectivity can be either structural or functional. Structural connectivity is based on landscape structure irrespective of the behavioral or biological attributes of a species relating with them (Kindlmann & Burel, 2008). In other words, it is based on corridors defined as relatively narrow strips of a landscape type that are different on both sides (Turner et al., 2001). On the other hand, the functional connectivity is defined as the degree to which the landscape facilitates or impedes species movement across patches in a habitat (Stevenson‐Holt et al., 2014). In other words, functional connectivity is a product of how landscape structure affect the dispersal or movement behavior of species (Puyravaud et al., 2017).

A related term “habitat connectivity” has been studied extensively. It is defined as the degree of functional connectivity between patches of optimal habitats for a specific species (Ayram et al., 2016; Puyravaud et al., 2017). This type of connectivity is based on the understanding that there is complex interaction between species movement (set by physiology and behavior) and landscape structure (set by landscape composition and configuration) which determines the ability of the species to move through the landscape (Goodwin & Fahrig, 2002).

However, as scientists try to stitch together the conservation areas using corridors, there is almost an equal but opposite force (called fragmentation) that disrupts connectivity, mainly caused by humans (McIntyre & Hobbs, 1999). Decrease in connectivity has been as a result of land‐use change and human‐induced landscape fragmentation and these factors have detrimental effects on the survival of many species (Cleary et al., 2017). Despite the old debate of whether a single large protected area is preferred to several small ones or not in conservation management (Williams et al., 2018), there is a common understanding among policy managers and scientists that habitats should be connected with each other. This is because habitat connectivity promotes seed dispersal, movement of taxa, response to climate change adaptation, and continuity of natural process (Pittiglio et al., 2012). Connectivity has become an important aspect for genetic connectivity mapping, designing movement corridors, and predicting environment changes (Spear et al., 2010).

Further, connectivity is vital for biodiversity conservation and management, particularly in protecting both endemic and endangered species (Liu et al., 2017). It also aids species to successfully reproduce in isolated patches (Cleary et al., 2017). More specific, connectivity as explained by Keeley et al. (2017) is critical for both natal and breeding dispersal of any organism. Natal dispersal is defined as movement of an animal from the areas where it is born to another area where it joins or attempts to join, the local breeding population. This kind of dispersal is critical for genetic diversity, demographic variability of metapopulations, recolonization, and range shifts, while breeding dispersal is movements by established adults to find breeding partners. It is also vital for maintenance of genetic diversity and reduce risks of inbreeding with close relatives (Keeley et al., 2017).

The influence of environmental variables on the movement and persistence of African elephants (*Loxodonta africana* herein called elephants) is relevant to policy makers, ecologists, and managers to develop balanced land‐use types in a landscape (Dutta et al., 2016). Elephants are known for traversing a mosaic of heterogenous landscapes (defined as the vegetation patches that vary in their composition and spatial arrangements (Beer & van Aarde, 2008)) in search of resources, that is, food and water (Gara et al., 2017). Consequently, elephants cover thousands of kilometers to maximize their quality forage and water intake. They are mixed feeders that incorporate varying amounts of grass and browse into their diet (Codron et al., 2006). They mostly browse leaves in the dry season and mainly feed on nutritious green grass in the early wet season (Kos et al., 2012). Elephants are the megaherbivores of savanna and habitat modifiers or ecological engineers as they significantly affect habitat conditions wherever they are found (Kohi et al., 2011). They are well known for their ability to change both structural and functional aspects of habitat that other species depend on (Codron et al., 2006). Economically, they are important for nonconsumptive tourism which in many African and Asian countries bring revenue to the countries (Barnes, 1996). Socially and culturally, elephants have for many years been the source of cultural ecosystem services for communities in Zambia, that is, Kuomboka ceremony conducted by Lozi King uses an elephant as a symbol of power (Kyando et al., 2017). However, management of elephants in Africa and specifically Zambia have not been without challenges, as elephants have been poached for their ivory or killed due to human–elephant conflicts (HECs) (Chase & Griffin, 2008; Lopes, 2014). As such, to effectively identify priority areas for management efforts, it is crucial to understand the interplay between elephant movement and landscape conditions such as suitable habitat areas, movement corridors, and patterns (Xu et al., 2017). Further, the identification of the movement patterns of elephants within corridors would help mitigate for habitat loss, reduce HECs and poaching as well as strengthening law enforcement (Williams et al., 2018).

Currently, there are several data types used to analyze habitat connectivity based on expert opinions, detection data, relocation data, pathway data, and genetic data (Zeller et al., 2012). As a result, a number of methods have been developed such as Classification and Regression Trees (CRTs), maximum entropy (MaxEnt), and circuits theory (Li & Hilbert, 2008; Merow et al., 2013; Phillips et al., 2006; Segurado & Araujo, 2004).

Therefore, it is against this background that the focus of the study is to predict suitable habitats, potential wet corridors, and barriers given a set of predictor variables using MaxEnt and linkage mapper. Elephants have been studied extensively (Druce et al., 2008); however, this is the first study that will try to understand how environmental factors affect their dispersal and persistence in Sioma landscape, particularly during the wet season. The study uses GPS telemetry data from the collared elephants to assess habitat connectivity by answering the following questions: (1) Which environmental variables and land‐cover class predict the movement of elephants during the wet season in Sioma Ngwezi landscape? (2) What is the wet season suitable habitat for elephants in Sioma Ngwezi landscape? (3) What are the major wet season movement corridors for elephants in Sioma Ngwezi landscape?

## 2 METHODS AND MATERIALS

### 2.1 Study area

**FIGURE 1:**
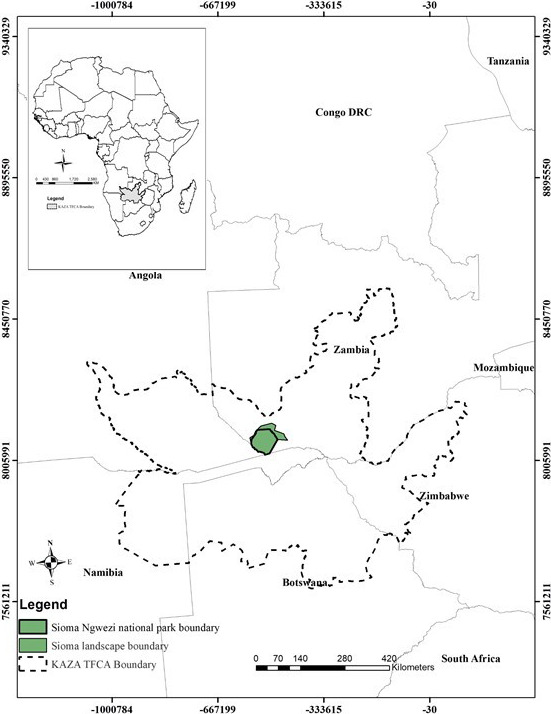
The location of the Kavango‐Zambezi Trans‐frontier Conservation Area (TFCA), and the Sioma landscape in western province of Zambia

Sioma Ngwezi national park (SNNP) is located in the southwest corner of Zambia, and it is the third‐largest protected area in Zambia with an estimated total surface area of 5,000 km^2^ (Chase & Griffin, 2008). West Zambezi Game Management Area (GMA) is the largest GMA in Zambia and it surrounds the park. Both SNNP and West Zambezi GMA (henceforth, referred to as Sioma landscape) had a stable elephant population prior to 1960s but were decimated due to illegal hunting which was exacerbated by the 25 year long Angolan Civil War (Chase & Griffin, 2008). The Sioma landscape is part of Kavango‐Zambezi Trans‐frontier Conservation Area (KAZA TFCA), which is the larger protected landscape that is shared among five countries, namely, Zambia, Zimbabwe, Namibia, Botswana, and Angola as shown in Figure 1. The study area is acting as a link between the source (Hwange, Chobe, and Bwabwata national parks) and the sink (the Greater Kafue and Luangwa ecosystems) of elephant movement in the KAZA TFCA (Roever et al., 2013).

The Sioma landscape predominantly comprises of broad floodplains, natural pools (dambos), water channel that dries up in the dry season, and linear dunes of sand (Burrough et al., 2015). The landscape has a tropical savannah climate with annual mean rainfall range from 600 mm to 800 mm, and a mean maximum temperature range of 27℃–30℃ and a mean minimum temperature range of 9℃–12℃ (Chase & Griffin, 2008). Additionally, the study area has relatively flat topography with the average elevation of between 970 m and 1,030 m above the sea level.

The landscape has heterogeneous vegetation typical for the Kalahari mainly composed of open grassy plains. The vegetation consists mainly of Leptochloa uniflora, Oplismenus hirtellus, Panicus heterostachum, Setaria homonyma, and terminalia species (Chase & Griffin, 2008).

### 2.2 Landscape data

The 100 × 100 km^2^ tiles of sentinel 2 (technically called Level 1C) satellite images with a ground sampling distance (GSD) of 10 m were downloaded. The downloaded images were sensed between May and June 2018 as they were less cloudy. We did image processing and classification using ENVI version 5.4. First, we made a seamless mosaic of images which was calibrated using radiometric calibration tool for easy and meaningful data analysis. Thereafter, quick atmospheric correction (QUAC) was used to correct for atmospheric effects on the seamless mosaic (Saini et al., 2016). Finally, maximum likelihood classification (MLC) algorithm was used to classify the spectral data into a thematic map. This algorithm was used because it performs better than other known parametric test in vegetation classification (Ganasri & Dwarakish, 2015). The algorithm uses Bayesian equation to assign the unknown measurement vector into a class with the highest probability of belonging by considering covariance within classes (Otukei & Blaschke, 2010). This algorithm used weighted distance (likelihood distance) to classify the entire seamless mosaic image for Sioma landscape using the following Bayesian equation:

D=ln(ac)‐[0.5ln(|covc|)]‐[0.5(X‐Mc)T(covc‐1)(X‐Mc)]

(Otukei & Blaschke, 2010)

The images were classified into the following four (4) broad classes:

- Built‐up places and bare land—This class comprised of areas that were exploited or disturbed by human beings such as settlements, agricultural areas, roads, airstrip, community schools, and cleared land for any purpose. These were then aggregated into one broad land‐cover class because elephants avoid such areas due to the presence of humans, especially during the day (Gaynor et al., 2018).
- Forest areas—These are areas with closed (above 70% crown cover) to open canopy (between 10 and 20% crown cover) Kalahari forests in the landscape. We used the FAO/UNEP Land Cover Classification System (LCCS) that defined open forests as “woodlands with short herbaceous layer” and closed forests as “continuous closed high forest with medium or low shrubs” (Jansen & Gregorio, 2003).
- Open water—This class consists of the water channels, streams, and the water pools usually used as source of water for wildlife within the landscape.
- Wooded grasslands—These are areas that grassy and sparsely populated trees and usually used by elephants as migrations links between forests or natural pools of water (Hensman et al., 2013).

We randomly generated 72 ground‐truthing points distributed equally among the 4 classes within the region of interest using ENVI. This means that there were 18 points to ground truth per class. Subsequently, ArcMap version 10.6.1 was used to convert the points into a shapefile format and converted the shapefile into KML file that was exported and loaded into Avenza map software on a Samsung phone. We then tracked all the points in the field. A confusion matrix was used to assess the overall accuracy, specificity, sensitivity, and Kappa statistic of the classified map. To reduce salt and pepper effect (mixture of classes) of traditional pixel classification, majority filter was used (Lu & Weng, 2007). This process filters and cleans the map but maintains both the integrity of the classes and details.

To create the elevation raster layer, we used Shuttle Radar Topography Mission Digital Elevation Model (SRTM DEM), an open‐access elevational data with the finest resolution of 30 m (Farr & Kobrick, 2000). To cover the whole study area, five tiles were mosaiced using ArcMap Mosaic tool. Thereafter, the layer was resampled to 10 m resolution and clipped to the right size of the study area.

Topographic Wetness Index (TWI) raster layer was created from SRTM DEM using System for Automated Geoscientific Analysis (SAGA) (Fisher et al., 2018) to show water flow from one area to another as well as soil moisture content. Both animals and plants are affected differently by TWI, and as such, it was included in the analysis as one of the explanatory variables.

Normalized difference vegetation index (NDVI) raster layer was created in ENVI software 5.6 (Nwaogu et al., 2017) using NDVI tool. NDVI is an indicator of relative biomass (i.e., healthy and photosynthetically active vegetation), which is calculated by dividing the difference of the near infrared (which vegetation strongly reflect) and red light (which is absorbed by vegetation) with the sum of near infrared and red light as shown in the formula: NDVI = (NIR − R)/(NIR + R) (Fitzgerald et al., 2018).

The following proximity raster was generated by first calculating the distance in meters from spatial points to the nearest dirt road and settlement; Proximity to dirt road raster was created from the elephants tracking points and their Euclidean distance from the road using inverse distance weighting (IDW) interpolation method. The method considers all the points independent of each other based on distance from each other or near features (Achilleos, 2011). Proximity to settlement raster layer was also created using IDW interpolation tool using distance of elephants tracking points to the legalized settlements in the landscape.

In 2017, 8 elephants from 4 groups (2 elephants from each group) in Sioma Ngwezi landscape were fitted with AWT ultrahigh‐frequency global position system (GPS) radio collars acquired from Africa Wildlife Tracking (Gara et al., 2017). These collars were fitted on 5 matriarchs and 3 bulls by experts from elephant connections organization. The collars were programmed to record elephant positions hourly in a quest to get detailed data of their movement in the landscape. Positional points from these collars had a ground precision of ±9 m. In 6 months (February–July 2018), a total of 30,454 points were recorded in the study area. In order to reduce both temporal and spatial autocorrelation which poses serious effect on ecological statistical models by inflating the predictive power of the model (Betts et al., 2009; Dormann et al., 2007), we randomly filtered the points and fixed to the 500 m distance between points as the minimum distance at which clusters are statistically less likely to form (Radosavljevic & Anderson, 2014). As a result, we used a total of 6771 data points in the model.

### 2.3 Statistical analysis

We used MaxEnt version 3.3.3K (available at http://biodiversityinformatics.amnh.org/open_source/maxent/) to predict wet suitable habitat areas for the elephants. This method can use either presence and absence data (if available) or presence‐only data to predict the habitat suitability of species. It has been the most preferred method with over 1,000 publications from 2006 to date (Merow et al., 2013; Morales et al., 2017). This has been possible merely for two reasons: First, the software outperforms other methods based on predictive accuracy, and second, MaxEnt is relatively easy to use compared to other methods (Merow et al., 2013). This explains why different governments, nongovernmental organizations and research groups have adopted to use MaxEnt for large‐scale and real‐world biodiversity mapping applications (Elith et al., 2011). However, MaxEnt has its own limitations, particularly for models predicated on presence‐only data. Like all models, they may fail to make accurate predictions if the researcher either assume incorrect predictor variables or ignoring other constraints (Harte et al., 2008). Elith et al. (2011) suggested two more limitations. First, models based on presence data only cannot approximate the actual probability of species habitat suitability due to lack of real absence points. However, such models give a relative probability of habitat suitability based on the frequency of presence and pseudo‐absence points. Second, selection bias (spatial autocorrelation) defined as unequal sampling of some areas in the landscape. This problem can be addressed by filtering the occurrence data (Phillips et al., 2009), even though, it does not completely get rid of spatial autocorrelation (Dormann et al., 2007).

The other method used in conjunction with MaxEnt in connectivity studies is linkage mapper tool built within Circuitscape. Within linkage mapper, there are two major tools, that is, circuit theory and least cost path tools. Circuit theory is a mathematically theoretical connectivity tool that estimates conductance (current flow) in a landscape. It is a model that represents random walker theory as it directly relates to the movement of species (McRae et al., 2008). On the other hand, least cost path is a graphical representation that assumes species movement or gene flow rates is direct related to the total cumulative resistance (resistance sum total value per pixel) along a single, optimal path between two core areas (Spear et al., 2010). Linkage mapper (circuit theory and least cost path) require a resistance surface when mapping corridors and pathways. Several quantitative methods for resistance surfaces and corridor visualizations have been developed in the last decade, based on expert opinion resistance values and empirical biological data. However, species movement remains a difficult behavior to observe and quantify mainly for two reasons: Firstly, when movement studies are conducted, the number of individuals being studied is usually small due to financial constrictions and secondly limited landscape accessibility (Zeller et al., 2012). Sometimes, the problem is escalated due to large gaps in the movement data from GPS collars. To avoid methodological subjectivity in movement studies, resistance surfaces created from habitat suitability model coupled with expert opinions are better in predictive power (Carroll et al., 2018; Zeller et al., 2012). Further, resistance to movement values is useful to give a quantitative estimate of how landscapes parameters affect species movements (Zeller et al., 2012). These maps depict the cost of movement in the landscape from one point to another as a functional landscape feature of that cell. In a similar sense, Singleton et al. (2002) defined resistance as the stepwise cost of moving through each cell for least cost‐based analysis, while McRae et al. (2008) defined the resistance surface as the relative probability of moving into the cell for circuit theory‐based analysis.

For these methods to work smoothly, collinearity must be assessed. The main problem with collinearity is inflating the parameter estimates produced by the model. Therefore, it is a requirement to assess collinearity of covariates prior to running any model irrespective of the method. Further, it is important to note that any approach to ecological modeling has little merit if the predictions are not assessed for their accuracy using an independent dataset (Fielding & Bell, 2002). All the methods for connectivity analysis require model validation threshold determined by area under receiver operating characteristic curve (AUC) and Kappa statistics (Tsoar et al., 2007). It has been generally accepted that AUC ranging from 0.7 to 1 better explained good model in terms of predictive performance (West et al., 2016). Cohen's Kappa (Cohen, 1960) commonly known as Kappa statistics define the accuracy of predictions, relative to the accuracy that might have resulted by chance alone. Kappa value ranges from −1 to +1 where +1 indicates a perfect agreement between predictions and observations while value of 0 or less indicates agreements no better than random (Allouche et al., 2006).

Before running maxent, collinearity was assessed for all the predictor variables in R version 3.5.2 (R Core Team, 2018), and when two covariates had pairwise correlation coefficient of |*r*| = 0.7, only one covariate of those pairs was selected for inclusion in the model (West et al., 2016). Of the 6771 elephant presence points prepared for the model, 3,771 (56%) presence points were used to train the model and the other 44% was used to validate the model. Thereafter, an optimal model (model that can explain most of the variation) was identified using MaxEnt. Since, species’ response to the predictor variables is very complex mathematically, we fitted nonlinear functions to resolve this complexity (Elith et al., 2011). Fitting nonlinear functions is done in the background by taking presence points and uncorrelated environmental predictors (i.e., land cover and elevation) as input for the software to work smoothly across the user defined grid cell (Merow et al., 2013). Just like in multiple regression where explanatory variables are transformed to fit complex models, MaxEnt does the same. The transformation functions used are referred to as features in machine learning and these are linear, product, quadratic, hinge, threshold, and categorical (Elith et al., 2011). Further, to produce meaningful results, MaxEnt binomial statistical equation (logistic regression) was used to extract background data points (pseudo‐absence points) from the user grid landscape combining it with the presence points. To avoid model overfitting, an optimal regularization gain function which penalizes the use of large values of the model parameters as well as ensuring that the empirical constraints are not fit too precisely was used (Phillips et al., 2006; Merow et al., 2013; Elith et al., 2011; Hijmans & Elith, 2017). Thereafter, the MaxEnt output called logistic multivariate environmental similarity surface (LMESS) that measures the similarity of any given point to a reference set of points, with respect to the chosen predictor variables was validated.

**FIGURE 2:**
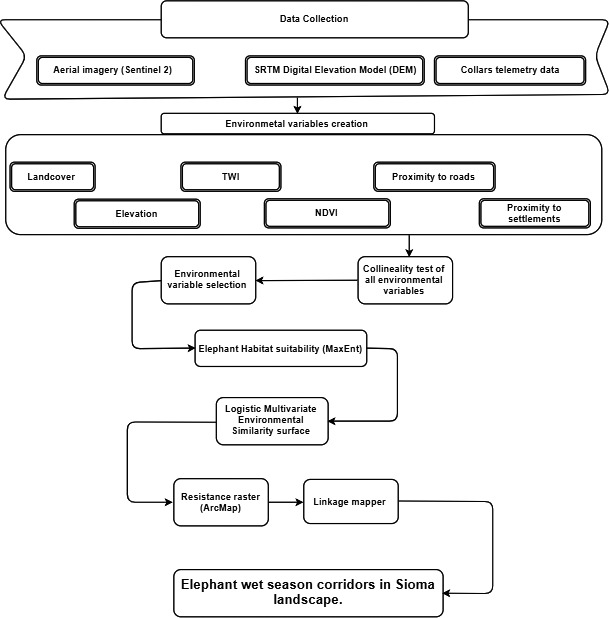
Flowchart of the major steps taken during the whole research period and the methodology to identify elephant corridors during the wet season in Sioma landscape. TWI is topographic wetness index and NDVI is normalized difference vegetation index

After generating the LMESS with MaxEnt, we imported the output into ArcMap for reclassification, thereby turning it into a resistance surface. In the context of Sioma landscape LMESS map, areas with highest relative probability of 1 were assigned lowest resistance value of 1, while areas with lowest probability (0) were assigned higher values of resistance of 10. In other words, the negative inverse of probability scale was used on the LMESS map to represent the resistance surface and then converted it to float file (Osipova et al., 2018). Fifteen core areas were identified using Kernel point density analysis (Osipova et al., 2018). Additionally, these areas have been reported by law enforcement officers as areas that elephant frequent during wet season. Subsequently, resistance raster together with linkage mapper was used for map out potential major wet season corridors for elephants in Sioma landscape. Linkage mapper used vector core areas and the resistance raster to identify least cost path between core areas as elaborated in the flowchart (Figure 2). The assumption here is, as the species move away from the core areas, cost‐weighted distance produces a map of total movement resistance accumulated. Linkage priority tool was used to visualize relative conservation priority of each corridor in the landscape. The tool uses multiple combinations of factors, namely, relative permeability of each linkage, proximity to other core areas, how central the linkage is to the entire network and expert opinions (incorporated when choosing predictor variables that were run in maxent) (Dickson et al., 2018).

## 3 RESULTS

After analyzing a total 6771 GPS data points from 8 elephants in Sioma Ngwezi landscape collected over a period of 6 months (February–July 2018), the following are the results:

### 3.1 Key environmental predictors

The MaxEnt model that explained most of the variation was identified with the use of beta multiplier of 1 with all the feature types. The corresponding feature types and regularization values were as follows: linear/quadratic/product:0.05, threshold:1, categorical:0.250 and Hinge:0.5. Of the six predictor variables, elevation (64%), land cover (13.5%), and NDVI (10.2%) were most important covariates with the highest relative contribution and permutation importance in the model. On the other hand, proximity to settlement (6.7%), proximity to dirt road (5.3%), and topographic wetness index (TWI) (0.4) were the least important as shown in Table 1.

**TABLE 1:** The variable contribution and permutation importance of each environmental variable of the optimal model with regularization value of 1 to identify the most important variables affecting the dispersal and persistence of elephants in Sioma landscape, Zambia

Variable	Percent contribution	Permutation importance
Elevation	56.6	64.0
Land cover	20.3	13.5
Normalized difference vegetation index (NDVI)	16.6	10.2
Proximity to settlement	3.6	6.7
Proximity to dirt roads	2.5	5.3
Topographic wetness index (TWI)	0.5	0.4

### 3.2 Land‐cover class

**FIGURE 3:**
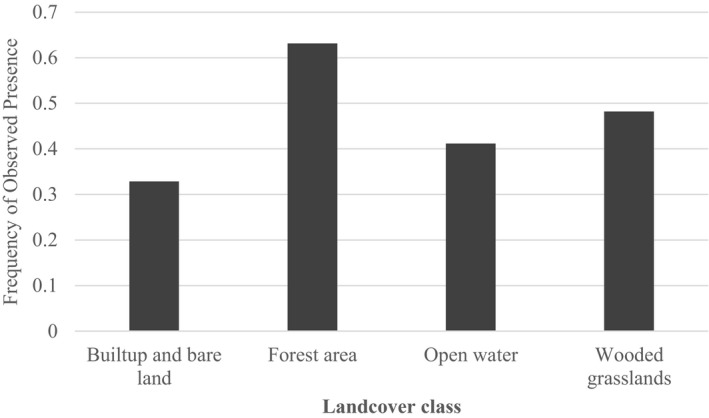
Distribution of presence points of collared elephants in four land‐cover classes in Sioma landscape Zambia. The frequency of presence points in the y‐axis is the ratio of elephant presence points to pseudo‐absence points in each land‐cover class

**FIGURE 4:**
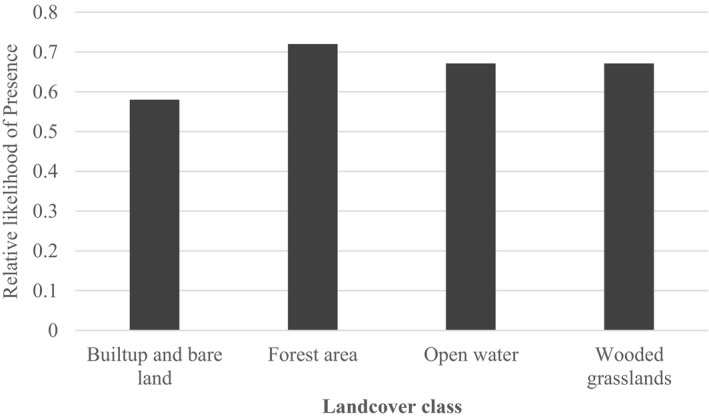
Relative probability of elephant presence in the four land‐cover classes in Sioma landscape based on a MaxEnt logistic transformation optimal model with all feature types (linear/quadratic/product:0.05, Threshold:1, categorical:0.250 and Hinge:0.5) and regularization value of 1. The optimal model had six predictor variables (elevation, land cover, NDVI, TWI, proximity to roads and proximity to settlements)

The land‐cover classification accuracy was assessed by using the confusion matrix based on the independent dataset generated from the field observations. The overall accuracy was 94.9% with Kappa statistics of 0.9 as shown in Table A1. Of the four land‐cover classes, forest areas had the highest frequency of observed presence points compared to wooded grassland, open water, and built‐up places as shown in Figure 3. Additionally, in terms of relative probability, elephants are more likely to be present in forested areas (0.71) than in wooded grasslands (0.67) or near open water (0.67) and near built‐up and open areas (0.59) during wet season as shown in Figure 4.

### 3.3 Wet season suitable habitats

**FIGURE 5:**
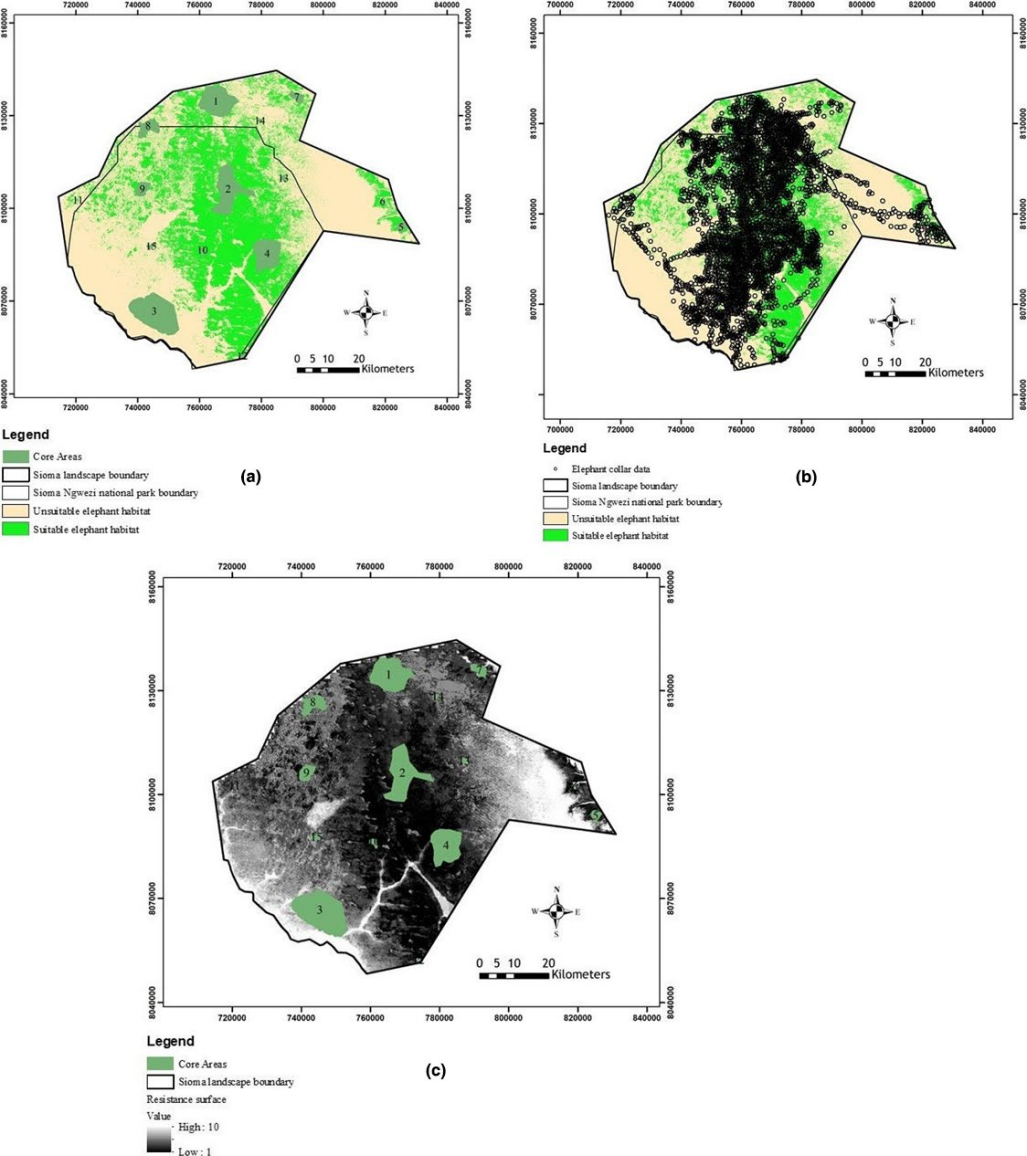
(a) Elephant suitable habitats are depicted in green patches while beige patches are unsuitable wet season habitats in Sioma landscape in Zambia. The classification into two classes was based on the use maximum training sensitivity and specificity threshold value of 0.54. Core areas were identified using Kernel point density analysis of presence points. (b) Showing an overlay of elephant movement points in grey on the habitat suitability map for Sioma landscape. (c) Illustration of landscape permeability for elephants in the landscape created from the negative inverse of the probability scale of the habitat suitability map. The darker areas have higher permeability compared to grey to white areas in the landscape. Core areas were identified using Kernel point density analysis of presence points

**FIGURE A1:**
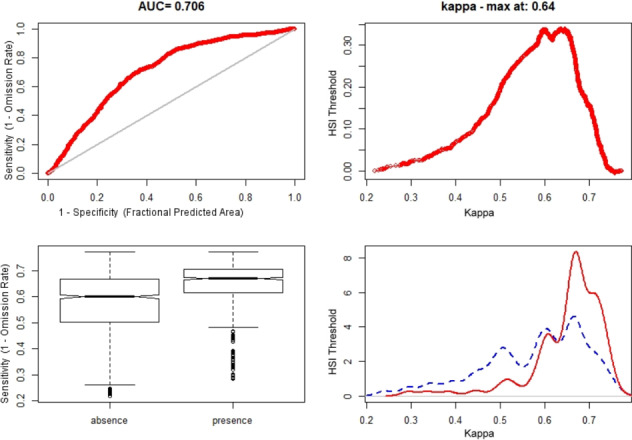
MaxEnt model validation shows how the model performed based on the under receiver operating characteristic curve (AUC) and Kappa values in the first two upper graphs. The red line in the left top figure shows the receiver operating curve in comparison with the black straight line with ROC value of 0.5. The ROC and Kappa values were calculated from actual telemetry data from elephant movement using confusion matrix based on sensitivity (false‐positive rate) and specificity (false‐positive rate) of both presence and pseudo‐absence (background data) points

The final LMESS model validation had AUC value of 0.706 and Kappa value of 0.64 as shown in Figure A1. The predicted elephant habitat classes for wet season, that is, suitable (green patches) and unsuitable (beige patches) habitats are shown in Figure 5a. The threshold used was maximum training sensitivity and specificity (Phillips et al., 2006; Merow et al., 2013; Elith et al., 2011; Hijmans & Elith, 2017). Additionally, an overlay of elephant presence points indicated majority of them fell in the predicted suitable habitats (Figure 5b). The negative inverse of the probability scale of the reclassified habitat suitability map (LMESS) is a resistance raster (Figure 5c) that was used in linkage mapper. The dark areas have lower resistance that allow permeability compared to the white areas and the identified core areas for elephants during the wet season are situated in areas with relatively lower resistance (Figure 5c).

### 3.4 Potential wet season movement corridors

**FIGURE 6:**
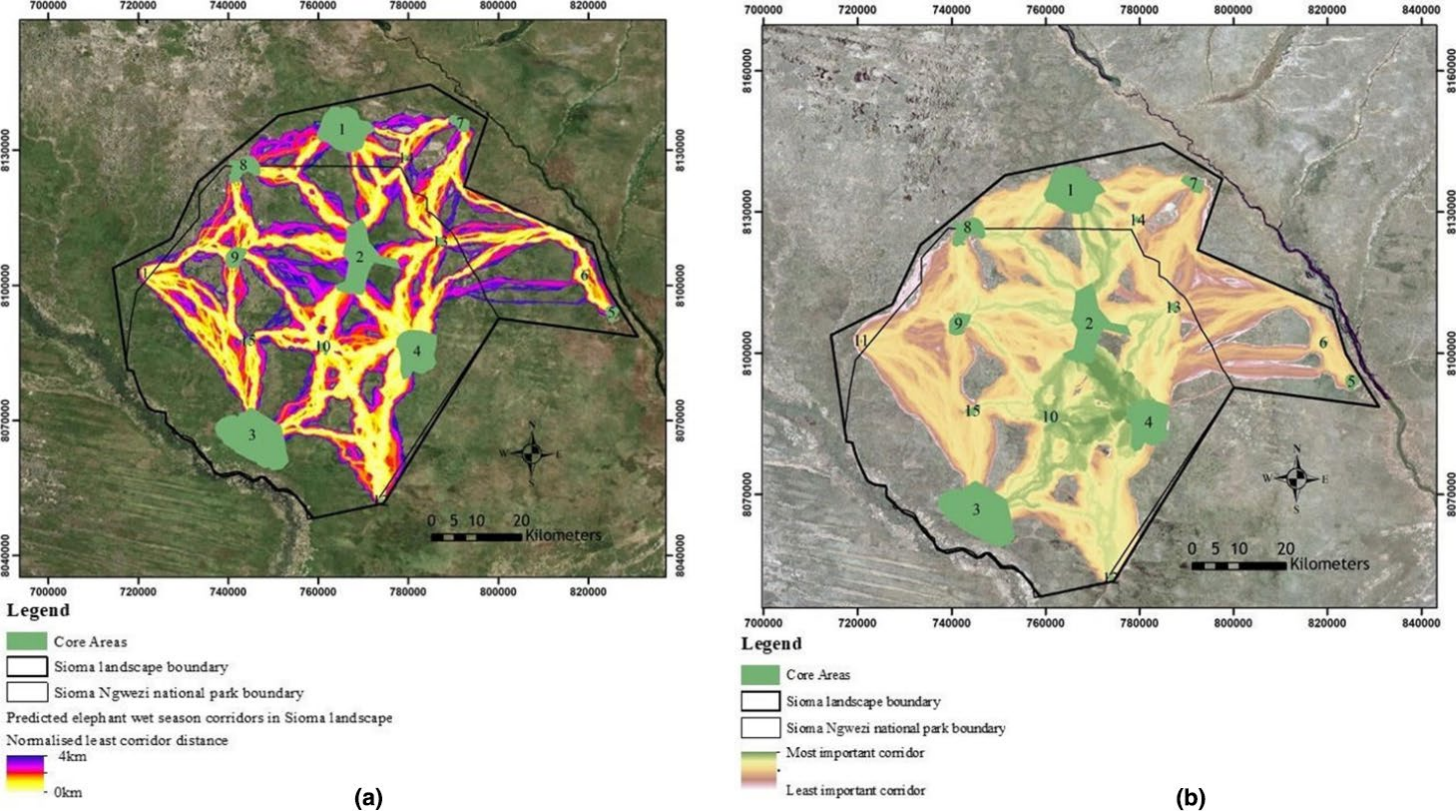
(a) Predicted potential elephant wet season corridors connecting the 15 core areas (green areas) in Sioma landscape. Bright yellow color shows least cost paths with higher conductance and less resistance in the landscape. The background image is Esri world imagery base map (Esri, 2009). (b) The light green corridors between core areas are illustrating the most important elephant wet season corridors in Sioma landscape. They were analyzed using linkage priority tool within Circuitscape. The background image is a mosaic of sentinel 2 images of the study area (mosaicked by the authors)

A total of 36 potential corridors were identified connecting 15 major core areas. The core areas numbered 1, 5, 6, 7, and 14 are in West Zambezi game management area (GMA) and are connected by corridors running within the GMA and into the park. Clearly, most of the elephant observed seem to avoid the lower south west part of the area due to human interface along the Cuando River but appear to prefer the central part of the park and the GMA (Figure 6a). Linkage priority output (Figure 6b) helps visualize the relative conservation priority of each corridor in a landscape. The results indicate linkages (light green corridors) between all core areas, except 5, 6, 11, and 12, as very important in facilitating smooth movement of elephants in Sioma landscape.

## 4 DISCUSSION

The results indicate that elephant distribution in the wet season in Sioma landscape is mainly predicted by three environmental factors, namely, elevation, land cover, and NDVI. On the other hand, proximity to settlements, proximity to dirt roads, and the topographic wetness index were the least important environmental variables in influencing the dispersal of elephants in the landscape. The elephants seem to prefer areas with elevation values ranging from 990 m to 1,020 m above sea level which is an area that runs from north to south centrally of the landscape (Figure 6). Elevation may have two‐pronged effect on the dispersal of elephants in the landscape, that is, indirectly or directly. The indirect one could be how elevation affects landscape diversity and configuration, soil and water dynamics which in turn affect the distribution of plant species that have direct impact on elephant presence in landscape (Hirzel et al., 2008). A study by Sanders and Rahbek (2012) highlighted the spatial elevation influence on the dispersal of different species and amalgamated the underlying causes of elevational diversity and dispersal as climate and productivity, source to sink dynamics, area, disturbance, geometric constraints, and evolutionary history. Other studies have also shown that elevation gradient does influence the dispersal and persistence of plants (West et al., 2016), as it has direct effect on local conditions of light, daily temperature amplitude, soil stability, and granulometry (Hirzel et al., 2008). The direct one would be that steep terrain affect their physical movement as evidenced in their avoidance of mountaineering (an energy‐expensive activity for the megaherbivores) (Lin et al., 2008; Wall et al., 2006).

Furthermore, the study results have also shown that forested areas play an important role in the distribution of elephants in the landscape. The area that has patches of forests also exhibited high NDVI values close to 1. Elephants seem to prefer these forest patches as they have the right mix of factors such as water availability as well as a productive, diverse, and abundant vegetation that meet their needs in the wet season. This was echoed by Chase and Griffin (2011) who also observed that for two concurrent elephant wet season surveys, herds mainly occurred within mixed savanna vegetation with numerous large pans with water. Loarie et al. (2009) shared similar results that in all the seasons of their study, the elephants were consistently selecting greener vegetation by utilizing vegetation with different phenology. However, the fact that elephant also spent time in grasslands may show that they are mixed feeders that consume a mixture of foliage, grass, and fruits (Codron et al., 2006). This kind of feeding makes it easier for them to traverse a mosaic of fragmented landscapes, at the same time meeting the energy demands of their physiology. Regrettably, late wet season has very high influx of elephants even in Sioma landscape that sometime over browse some vegetation types that form most of their diet (Ihwagi et al., 2010). Aarde and Jackson (2006) termed this phenomenon as “elephant problem” because high numbers of elephants in a localized place usually is believed to degrade vegetation to the detriment of other species that also depend on the same vegetation. This problem is not exacerbated by shortage of grazing or browsing areas of elephants, but their strong preference on certain plants, that is, *Sclerocarya birrea*, *Terminalia sericea*, *Combretum apilatum* (Seloana et al., 2018). Furthermore, in a study conducted by Schmitt et al. (2018), it was found that elephants effectively used their olfactory cues to differentiate odor profiles of palatable and the unpalatable plant species between forest patches. Thus, provision of functional corridors is one of the remedies that would allow movement of elephants between these patches in search of palatable plants and grasses.

Proximity to settlements seem not to affect the dispersal of elephants in the landscape as they may have gotten accustomed to foraging around settlements and possibly their affinity to crop‐raiding (Orrick, 2018). This adaption in elephants is highly associated with human–elephant conflicts (HECs). In Zambia, elephants represent the highest human–wildlife conflicts especially in game management areas (Youldon et al., 2017). As a result of HECs, a number of methods have been used in Africa and Asia to abate crop‐raiding ranging from using beehives, chili powder, elephant dung, and electric fences to traditional ones such as firecrackers, dogs, watch towers, drums, and delineating buffer zones that support the coexistence of both communities and wildlife (Davies et al., 2011; King et al., 2011; Ngama et al., 2016; Pozo et al., 2019; Scheijen et al., 2019). All these deterrent methods are useful, but electric fencing is more successful albeit it being expensive in setting. However, care should be taken in positioning these fences as they affect the movement of many other species move in the landscape (Druce et al., 2008; Osipova et al., 2018). Similarly, proximity to dirt roads seems not to deter elephant movement in Sioma landscape. This may be attributed to dirt roads not posing any danger to elephants foraging near and crossing the roads especially that our study area is in a rural area with little traffic. These dirt roads are not as busy as the public paved road (Blake et al., 2008).

Consistent with other studies (Blake et al., 2008; Loarie et al., 2009; Orrick, 2018), elephants may chose habitat utilization in protected areas, relatively fragmented landscapes and at times near settlements and roads. Knowing the extent and locations of suitable habitat would help to protect and manage elephants effectively and efficiently (Williams et al., 2018). However, as indicated before, a suitable habitat for any species is affected by an array of environmental variables at varying scales. The habitat suitability model was at a landscape scale and as such land use, land cover, and topography are the most important variables that influenced it (Bradley et al., 2012 *)*. Clearly, there is a reduction of suitable habitat along the Cuando River (south west corner of the landscape) due to the heavily settled riverbank and cleared agriculture fields (Figure 5). The predicted suitable habitats significantly support the elephant movement in the landscape during the wet season as shown in Figure 6. Although the collected data did not cover dry season, elephants seem to withdraw from this landscape to Kwando river basin during dry season as the area is characterized with relatively high temperatures and most of the water dambos dry out (Chase & Griffin, 2011).. Additionally, the remnant vegetation patches are usually burnt by the frequent summer fires (Chase & Griffin, 2008). As a result, wet season elephant habitat is crucial in as far as their dispersal and landscape utilization is concerned. Most importantly, the landscape has no barriers that would significant impede the movement of elephants.

To know how permeable the landscape was in the wet season was paramount for several reasons. Firstly, elephants navigate in the landscape while maximizing on their feeding, seeking protection, and water, and by analyzing their movement, we can understand the functional connectivity of the elephant out of the elephant's perspective. Secondly, understanding the landscapes functional connectivity would help conservationists to curb poaching as law enforcement officers can direct their patrols around the identified corridors. And thirdly, the identified corridors will help curb HECs by managing these corridors (Williams et al., 2018). Roever et al. (2013) summed it up by proposing that restoration of elephant connectivity in southern Africa may reduce local impacts, reduce the need for elephant culling, and eventually stabilize elephant numbers in the region. Although habitat connectivity among organisms differ, Epps et al. (2011) stated that elephant presence is highly positively correlated with the richness of other mammals with weight greater than 45 kg. Given the understanding that elephants have a wider home range between 10 km^2^ and 21,000 km^2^ than most other animals (Gara et al., 2017), the focal species linkage mapping approach developed in this project would be a conduit through which other species will be guaranteed protection as well. This concept was reiterated by Roever et al. (2013) referring to elephants as umbrella or flagship species if well managed would benefit other species too.

Finally, Sioma landscape is strategically located in the Kavango‐Zambezi TFCA especially that it is acting as a link between the source (Hwange, Chobe, and Bwabwata national parks) and the sink (the Greater Kafue and Luangwa ecosystems) of elephant movement in the KAZA TFCA. Recently, Roever et al. (2013) estimated that close to 60% of the African elephants are found in the protected areas of southern Africa particularly the big five national parks (Chobe, Kafue, Luangwa, Lower Zambezi, and Hwange). Consequently, management of the predicted corridors in Sioma landscape would support movement of elephants from north to south of the TFCA as a single entity. However, identification of the functional elephant corridors in the landscape is not an end in itself but may require both political will and knowledge‐based management to produce the desired outcome of their functionality.

## 5 CONCLUSION

The study utilized elephant telemetry movement data to identify functional connectivity and core areas in a region during the wet season. Though costly, it is the most reliable method of studying elephant distribution in the landscape. The use of habitat suitability models (i.e., MaxEnt) is an effective objective way of creating resistance raster that is important in species movement studies. The study successfully achieved to identify the most influential environmental variables and the land‐cover class that predict the movement and persistence of elephants in Sioma landscape. Further, suitable wet season habitats and their potential wet corridors were also identified in the landscape. Of conservation relevance, the maps have shown predicted areas that support elephant movement in the wet season. This study has also shown corridors that may be managed to reduce HECs, poaching, and strengthen law enforcement by patrolling areas with high probability of finding elephants. Therefore, we recommend the findings of this study to the department of national parks and wildlife (Zambia) to follow up with action particularly in creating land‐use plans in the landscape that will recognize the identified elephant corridors. However, this research will be more complete if dry season movement patterns are also studied and then compared. It will help management to have a complete understanding on how seasonality affects the distribution of elephants in the landscape and possibly alleviate the problems associated with them.

## CONFLICT OF INTEREST

None declared.

## AUTHOR CONTRIBUTIONS

**Doubt Chibeya:** Conceptualization (lead); data curation (lead); formal analysis (lead); funding acquisition (lead); investigation (lead); methodology (equal); project administration (lead); resources (equal); software (equal); supervision (equal); validation (equal); visualization (equal); writing–original draft (equal); writing–review and editing (equal). **Heather Wood:** Conceptualization (supporting); data curation (supporting); formal analysis (supporting); funding acquisition (supporting); investigation (supporting); methodology (supporting); project administration (supporting); resources (equal); software (equal); supervision (equal); validation (equal); visualization (equal); writing–original draft (equal); writing–review and editing (equal). **Sara A. O Cousins:** Conceptualization (supporting); data curation (supporting); formal analysis (supporting); funding acquisition (supporting); investigation (supporting); methodology (supporting); project administration (supporting); resources (equal); software (equal); supervision (equal); validation (equal); visualization (equal); writing–original draft (equal); writing–review and editing (equal). **Kerryn Carter:** Conceptualization (supporting); data curation (supporting); formal analysis (supporting); funding acquisition (supporting); investigation (supporting); methodology (supporting); project administration (supporting); resources (equal); software (equal); supervision (equal); validation (equal); visualization (equal); writing–original draft (equal); writing–review and editing (equal). **Moses Amos Nyirenda:** Conceptualization (supporting); data curation (supporting); formal analysis (supporting); funding acquisition (supporting); investigation (supporting); methodology (supporting); project administration (supporting); resources (equal); software (equal); supervision (equal); validation (equal); visualization (equal); writing–original draft (equal); writing–review and editing (equal). **Henry Maseka:** Conceptualization (supporting); data curation (supporting); formal analysis (supporting); funding acquisition (supporting); investigation (supporting); methodology (supporting); project administration (supporting); resources (equal); software (equal); supervision (equal); validation (equal); visualization (equal); writing–original draft (equal); writing–review and editing (equal).

### OPEN RESEARCH BADGES

This article has been awarded <Open Data, Open Materials> Badges. All materials and data are publicly accessible via the Open Science Framework at https://doi.org/10.1002/ece3.8177.

## Data Availability

Due to the sensitive nature of the location data of elephants used in this research, data are only available for internal use for the Department of National Parks and Wildlife in Zambia and their partners. For external researchers, ethical approval may be obtained via formal application to the Director, Department of National Parks and Wildlife in Zambia. However, interested parties are advised to contact the corresponding author (doch3117@student.su.se) to discuss the application.

## APPENDIX 1

**TABLE A1:** A confusion matrix table was used to calculate the overall accuracy and kappa value of the classified satellite imagery based on field‐validated data

Land cover classification		Ground truthing/Reference Data							
		Built‐up, Bare land and Agricultural land	Wooded grasslands	Forested areas	Water	Row total	Row total in (%)	User accuracy	Commission error
Classified data	Built‐up, Bare land and Agricultural land	19	0	0	0	**19**	24.4%	100.0%	0.0%
	Wooded grasslands	1	17	2	0	**20**	25.6%	85.0%	15.0%
	Forested areas	0	1	18	0	**19**	24.4%	94.7%	5.3%
	Water	0	0	0	20	**20**	25.6%	100.0%	0.0%
	**Column total**	**20**	**18**	**20**	**20**	**78**	Overall Accuracy 94.9		
	Marginal total (%)	25.6%	23.1%	25.6%	25.6%				
	Producer accuracy	95.0%	94.4%	90.0%	100.0%	–			
	Omission error	5.0%	5.6%	10.0%	0.0%				
	Agreement	19	17	18	20	74			
	Chance	6.2%	5.9%	6.2%	6.6%	20.0%			
		4.9	4.6	4.9	5.1	19.5			
		Kappa Value 0.9							

Note

Field data were collected from four land‐cover classes. The kappa value was 0.9, and overall accuracy was 94.9.
